# Hope for the Best or Prepare for the Worst? Towards a Spatial Cognitive Bias Test for Mice

**DOI:** 10.1371/journal.pone.0105431

**Published:** 2014-08-19

**Authors:** Vanessa Kloke, Rebecca S. Schreiber, Carina Bodden, Julian Möllers, Hanna Ruhmann, Sylvia Kaiser, Klaus-Peter Lesch, Norbert Sachser, Lars Lewejohann

**Affiliations:** 1 Department of Behavioural Biology, University of Münster, Münster, Germany; 2 Otto Creutzfeldt Center for Cognitive and Behavioral Neuroscience, University of Münster, Münster, Germany; 3 Division of Molecular Psychiatry, Laboratory of Translational Neuroscience, Department of Psychiatry, Psychosomatics and Psychotherapy, University of Würzburg, Würzburg, Germany; 4 Behavioral Biology, University of Osnabrück, Osnabrück, Germany; Alexander Fleming Biomedical Sciences Research Center, Greece

## Abstract

Cognitive bias, the altered information processing resulting from the background emotional state of an individual, has been suggested as a promising new indicator of animal emotion. Comparable to anxious or depressed humans, animals in a putatively negative emotional state are more likely to judge an ambiguous stimulus as if it predicts a negative event, than those in positive states. The present study aimed to establish a cognitive bias test for mice based on a spatial judgment task and to apply it in a pilot study to serotonin transporter (5-HTT) knockout mice, a well-established mouse model for the study of anxiety- and depression-related behavior. In a first step, we validated that our setup can assess different expectations about the outcome of an ambiguous stimulus: mice having learned to expect something positive within a maze differed significantly in their behavior towards an unfamiliar location than animals having learned to expect something negative. In a second step, the use of spatial location as a discriminatory stimulus was confirmed by showing that mice interpret an ambiguous stimulus depending on its spatial location, with a position exactly midway between a positive and a negative reference point provoking the highest level of ambiguity. Finally, the anxiety- and depression-like phenotype of the 5-HTT knockout mouse model manifested - comparable to human conditions - in a trend for a negatively distorted interpretation of ambiguous information, albeit this effect was not statistically significant. The results suggest that the present cognitive bias test provides a useful basis to study the emotional state in mice, which may not only increase the translational value of animal models in the study of human affective disorders, but which is also a central objective of animal welfare research.

## Introduction

While cognitive factors can be of fundamental importance in determining emotional experiences, emotional information can also selectively influence cognitive processes, including attention, memory, and judgment [1]–[3]. For instance, people in negative affective states show enhanced attention to threatening stimuli, retrieve negative memories, and make negative judgments about future events or ambiguous stimuli more than people in positive affective states [1], [4]–[9]. Such emotion-mediated cognitive biases are assumed to play an important role in the development, maintenance, and recurrence of depression and anxiety disorders [10]–[13]. Furthermore, their modification has been suggested as an innovative strategy for the treatment of the illness [14]–[16].

In animals, the cognitive component of emotion has long remained relatively unexplored and animal affect has traditionally been inferred from behavioral and physiological measures such as anxiety-like behavior or hypothalamic-pituitary adrenal (HPA) axis activity [1], [2]. However, as the use of such measures is limited, the concept of cognitive bias has been suggested as a promising new tool to assess emotional valence in animals [1]. The seminal work in this field was carried out by Harding and coworkers, who introduced a judgment bias test for rats, in which the propensity for rather positive or rather negative expectations was assessed by the response to affectively ambiguous stimuli [17]. Rats were trained to press a lever when they heard a tone associated with a positive event (food-delivery) and to desist from pressing a lever when they heard another tone in order to avoid a negative event (burst of white noise). Once trained on this discrimination task, rats were then exposed to non-reinforced tones of intermediate frequencies between the food-delivery and noise-avoidance tone. In line with studies in humans, rats in a putatively negative emotional state - induced by unpredictable housing conditions - showed behavior indicating a reduced anticipation of a positive event, i.e. they responded slower and tended to show fewer responses to the ambiguous tones than control rats [17]. Since 2004, cognitive bias test paradigms have not only been further validated for rats [17]–[23] but also for a wide range of other animal species, including monkeys [24], birds [25]–[27], dogs [28], [29], several farm animals [30]–[32] and even invertebrates [33], [34]. The fact that mice are the premier mammalian model system in preclinical neuropsychological research calls for reliable methods to assess affective states in this species as well. If cognitive biases can be assessed in mice, this may fundamentally increase the translational value of many mouse models and consequently may help to extend and refine the understanding of human emotional disorders [21]. A promising approach in this field has already been made by Boleij and associates, who established a cognitive bias test, in which two distinct odors predicted either a palatable or an unpalatable food reward and the reaction of the mice to a mixture of both odors was used as a measure of their judgment bias [35]. However, since the task requires stable odor discrimination, which one of the two tested mouse strains in the study failed to exhibit, further investigations are still needed.

In the present study we wanted to develop a novel cognitive bias test for mice involving another sensory modality - spatial perception - and to apply this test paradigm in a pilot study to a genetically modified mouse model of anxiety- and depression-related behavior. We used a spatial judgment task in which mice were trained to expect a positive event (access to the home cage) in one location and a negative event (air-puff) in another location, to determine how mice in a relatively positive or negative emotional state respond to an ambiguous stimulus of intermediate spatial location. We chose spatial location as a discriminatory stimulus because it has pronounced salience in cognitive tasks for many animals [18], [21], [36], [37] and is of high ecological relevance: Mice live in burrows ranging from simple straight tunnels to complex systems [38] and learning and remembering of places is necessary to locate food resources, potential predators, escape routes, immediate kin, or territory boundaries [39], [40]. Particularly important is that not only locations in the environment are remembered, but also contents of those locations or important events that occurred there [41].

Our aims were first, to validate that the novel test paradigm can assess different expectations about the outcome of an ambiguous stimulus (experiment I). We hypothesized that mice anticipating a positive event would differ in their behavior towards an unfamiliar stimulus than mice anticipating a negative event. Second, we wanted to investigate the applicability of spatial location as a discriminatory stimulus (experiment II). We expected that the mouse would interpret an ambiguous stimulus depending on its spatial location, with a position exactly midway between a positive and a negative reference point provoking the highest level of ambiguity. Finally, the test paradigm was used in a pilot study with serotonin transporter (5-HTT) knockout mice (experiment III), which have proven to be a powerful tool to study the role of altered serotonergic activity in emotion regulation [42], [43]. Homozygous 5-HTT knockout mice, in which 5-HTT function is completely absent, display a range of phenotypic changes, in particular increased anxiety-related behavior, decreased exploratory locomotion, depression-related behaviors as well as altered stress coping abilities [43]–[50]. Heterozygous 5-HTT knockout mice, which display reduced 5-HTT expression of about 50 %, are often similar to wildtype mice or develop phenotypic alterations only under more challenging environmental conditions [51]. In this pilot study we wanted to test the hypothesis that the anxiety- and depression-like phenotype of the 5-HTT knockout mouse model manifests also in a negative distorted interpretation of ambiguous information.

## Materials and Methods

Three experiments were conducted to establish a cognitive bias test with mice using a spatial judgment task. Please note: To increase comprehensibility, experiments are presented in reverse chronological order, i.e. experiment III was performed first, followed by experiment II and I. Procedural changes (e.g. apparatus, trial intervals) are due to optimization of the test over time with the apparatus and procedures in experiment I representing the latest state of development.

### Animals and general housing conditions

Experiment I was conducted with 14 female C57BL/6N mice (Charles River, Sulzfeld, Germany), which were about 6 months old at the start of the experiment. Tests in experiment II were performed with 36 female C57BL/6J mice (Harlan Laboratories, Venray, The Netherlands), which were about 2 months old at the start of the tests. Experiment III was carried out with 36 female wildytpe (+/+), heterozygous (+/−), and homozygous (−/−) 5-HTT knockout mice (Bengel et al. 1998), backcrossed into a C57BL/6J genetic background for >10 generations, which originated from the internal stock of the Department of Behavioural Biology at the University of Münster, Germany. Mice were about 3–4 months old at the start of the experiments. Genotypes were identified by gel electrophoresis of DNA fragments of either 225 bp (5-HTT +/+), 272 bp (5-HTT −/−), or both (5-HTT +/−).

All mice were housed in same-sex groups of two to five individuals in transparent standard Makrolon cages type III (42×27×16 cm) with sawdust as bedding material (Allspan, Höveler GmbH & Co. KG, Langenfeld, Germany), a paper towel as nesting material, and food (1324, Altromin GmbH, Lage, Germany) and water provided *ad libitum*. In experiment III, mice were housed in mixed-genotype groups. Mice were additionally fed oat flakes once a week (Fortin GmbH & Co. KG, Düsseldorf, Germany). The colony room was maintained at a 12 h light/dark cycle with lights on at 08:00 a.m. and an average temperature of 22±2°C and humidity at 45%±15.

### Ethics Statement

The present work was carried out in strict accordance with current regulations covering animal experimentation in Germany and the EU (European Communities Council Directive 86/609/EEC). All experiments were announced to the local authority (North Rhine-Westphalia State Agency for Nature, Environment and Consumer Protection, LANUV) and were approved by the ‘Animal Welfare Officer’ of the University of Münster (reference number experiment I: 8.84–02.05.20.11.049, experiment II: 8.84–02.05.20.11.119, experiment III: 8.87–51.05.20.10.052). All efforts were made to minimize suffering.

### Experimental design

#### Experiment I

Aim of this experiment was to show that mice trained to expect a positive event differ in their behavior towards an unfamiliar stimulus in comparison to mice trained to expect a negative event. For optimistically-trained mice (n = 7) two reference locations within a maze could be used to escape the brightly illuminated test apparatus. For pessimistically-trained mice (n = 7) the same two reference locations predicted punishment in form of an air-puff when being entered. After training, optimistically- and pessimistically-trained mice were tested for their behavior towards an unfamiliar location, located midway between the two reference locations.

#### Experiment II

Aim of this experiment was to show that the mouse interprets an ambiguous location within the maze depending on its spatial location. For this purpose, three groups of mice were trained similarly to discriminate between a positive and a negative reference location, either predicting access to the home cage or punishment in form of an air-puff. Afterwards mice were tested for their behavior towards one of three ambiguous locations, which were distributed at intermediate points between the two reference locations: Mice of the central group (CE, n = 12) were tested for their behavior towards a central probe arm located halfway between the positive and the negative reference location; for mice of the near-negative group (NN, n = 12) the ambiguous probe location was located halfway between the central probe arm and the negative reference location; for mice of the near-positive group (NP, n = 12) the ambiguous probe location was located halfway between the central probe arm and the positive reference location.

#### Experiment III

This pilot study assessed the applicability of the test paradigm for the evaluation of cognitive bias in 5-HTT knockout mice. 5-HTT+/+, 5-HTT+/−, and 5-HTT −/− mice (each n = 12) were trained similarly to discriminate between a positive and negative reference location, either predicting access to the home cage or punishment in form of an air-puff. Subsequently, mice of all three genotypes were tested for their behavior towards an ambiguous location, located midway between the two reference locations.

### Procedure

#### Experiment I

**Figure 1:**
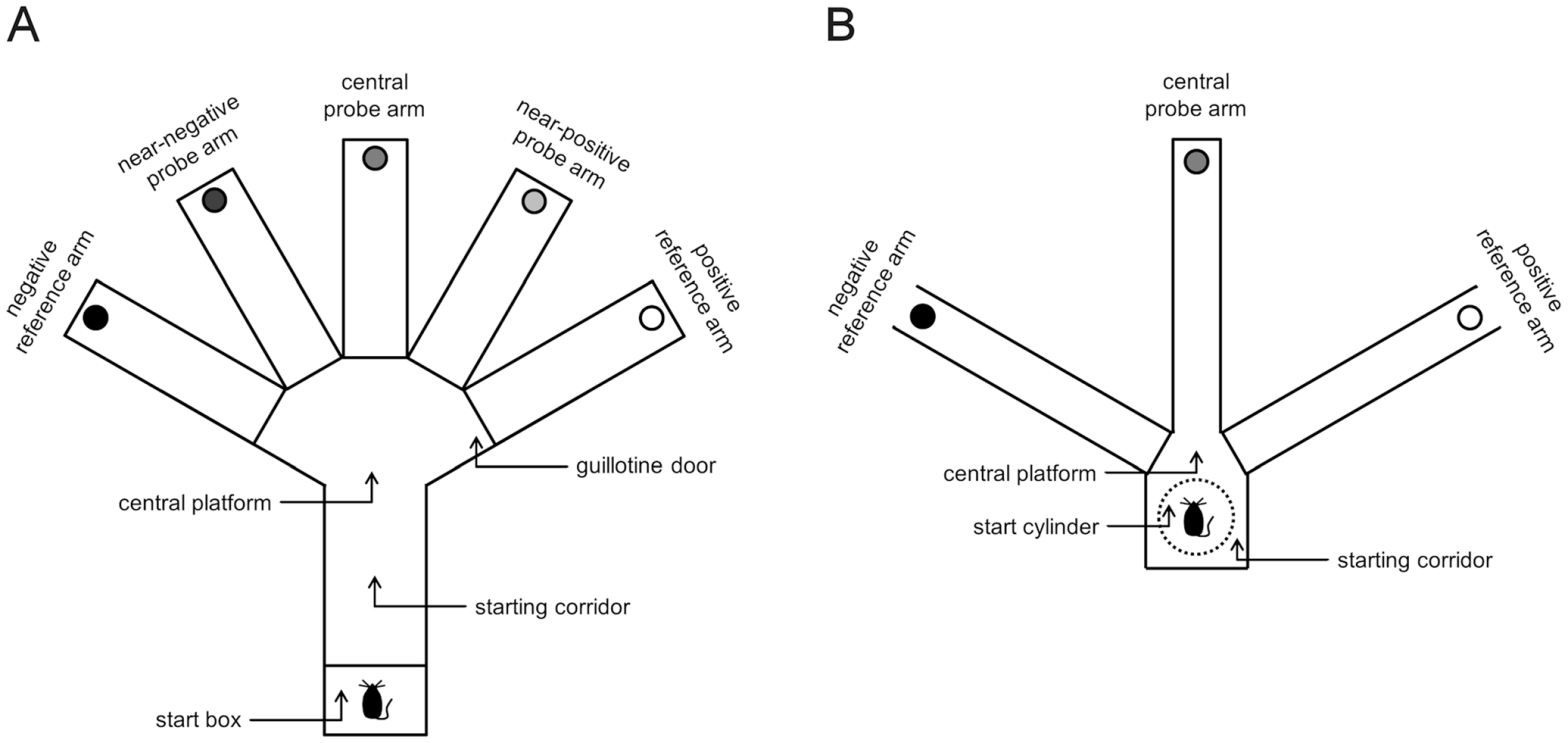
Cognitive bias test apparatus. (**A**) Schematic diagram of the apparatus used in experiment I and II displaying the start box, the starting corridor, the central platform, the positive and negative reference arm, the three probe arms, and the sliding doors. In experiment I both reference arms had either a positive or a negative outcome and only the central probe arm was used for the probe trial. (**B**) Apparatus used in experiment III with the start cylinder positioned in the starting corridor, the central platform, the positive and negative reference arm, and the ambiguous probe arm. Unused arms were closed by reversing them so that their closed end functioned as barrier. Please note: The position of the positive and negative reference location in experiment II and III was counterbalanced between individuals.

*Apparatus*: The cognitive bias test apparatus (Fig. 1A) was made of dark grey PVC and was positioned on a white coated board made of plywood that was elevated 120 cm above the ground. The apparatus was open at the top and consisted of a starting corridor (37×15×15 cm) leading to a central area from which five equidistantly spaced arms (30×8×15 cm) radiated. The two reference arms (‘positive’  =  rewarded or ‘negative’  =  aversive) were positioned 120° from each other, while the three probe arms were positioned at equidistant angles between the two reference locations, each separated by 30°. Hence, one probe arm (‘central’) was located midway between the two reference locations, and the other two probe arms (‘near-negative’ or ‘near-positive’) were halfway between the central probe arm and each reference arm. In experiment I, only three arms of the apparatus were used, namely the two reference arms and the central probe arm. The access from the central platform to each arm could be regulated by manually operated sliding doors. An additional sliding door was installed in the closed end of the starting corridor, forming a start box (10×15×15 cm), in which mice were placed before the beginning of each trial.

In each trial the door to just one arm was opened, while the doors of the four unused arms were closed. There was a hole of 3 cm in diameter embedded in the floor at the outer end of each arm. For optimistically-trained mice, a wire-mesh tunnel connected the hole in both reference arms (‘positive’  =  rewarded) via a specially constructed cage-lid with the home cage of the tested animal. Thus, mice could escape from the brightly lit test apparatus into their home cage by entering the hole. For pessimistically-trained mice, the hole in both reference arms (‘negative’  =  aversive) was closed by a blind wire-mesh tunnel equipped with a tube that was connected to a manually operated air pump. Whenever the mouse touched the hole with any part of its body as seen from above, an air-puff was released by the experimenter who observed the behavior of the mouse via a camera mounted above and attached monitor. During probe trials, the hole was closed by a blind wire-mesh tunnel without any tube or tunnel to the home cage connected. No additional landmarks were provided, thus it was not possible for the mice to discriminate visually between the outcomes of the trial before directly looking into the respective hole. Moreover, the home cage of the test animal was positioned directly beneath the center of the test apparatus in training trials with access to the positive arm, thus mice could not use olfactory cues for orientation.

The test apparatus was positioned in a room different from that in which the mice were housed and the test equipment was cleaned with 70% ethanol between subjects. The movements of the animal were recorded and analyzed by the video tracking system ANY-maze (Version 4.75, Stoelting Co., Wood Dale, USA).

*Training*: Mice were trained over a period of 3 days for a total of 13 trials (day 1: 5 trials, day 2: 6 trials, day 3: 2 trials). Trials on each day were conducted in sessions of 2 or 3 trials each, interspersed by a break of at least 30 minutes. In each training trial mice had either access to the left or the right reference arm with both arms being presented alternately.

A training trial always started with placing the mouse in the start box with the sliding door closed for a start interval of 30 s. Once the 30 s had elapsed, the sliding door was opened and the mouse could freely explore the apparatus. Training trials for optimistically-trained mice ended with the mouse entering the hole to the home cage or reaching a cut-off point after 180 s (trial 1+2) or 45 s (trial 3–13). If a mouse did not enter the hole within the allotted time, it was gently forced to enter to go back to its home cage. Optimistically-trained mice were allowed to stay in their home cage for 45 s before the next trial started or remained in the home cage in the event that no further trials followed. Training trials for pessimistically-trained mice always ran the maximal time of 180 s (trial 1+2) or 45 s (trial 3–13). Whenever the mouse reached the hole with any part of its body, an air-puff was released. If pessimistically-trained mice did not reach the hole within the regular test time, no additional special treatment followed. Importantly, all pessimistically-trained mice experienced the punishment by an air-puff at least once during the training period. After the end of the trial the mouse was directly placed in the start box for 30 s prior to the next trial. If no further trials followed, the mouse was also placed in the start box for 30 s and subsequently taken out of the start box and returned to its home cage.

*Probe trial*: After the last training trial on day 3, mice were tested for their behavior towards an unfamiliar location in a probe trial on the central probe arm. Similar to the training trials, they spent 30 s in the start box before gaining access to the whole test apparatus, this time with the sliding door to the central probe arm opened and the four unused arms closed. The probe trial lasted 60 s and the parameters measured were the latency to reach the hole, the latency to reach the arm, and the time spent at the hole.

#### Experiment II

*Apparatus:* The same apparatus was used as in experiment I, this time with all five arms being operational. Moreover, the two reference arms had a different outcome, i.e. one reference arm (‘positive’  =  rewarded) led to the hole connected to the wire-mesh tunnel leading to the home cage, while the other reference arm (‘negative’  =  aversive) led to the hole with the air pump connected.

*Training:* Mice were trained over a period of 4 days for a total of 21 trials to discriminate between the positive ( =  rewarded) and negative ( =  aversive) reference location. The increased number of training trials and days in comparison to experiment I were chosen because of the higher complexity of the learning task. Trials on one day were conducted in sessions of 2 or 3 trials each, with the two sessions being interspersed by a break of approximately 2 hours. In each training trial, mice had access to either the positive reference arm or the negative reference arm. The position of the positive and negative reference arm was balanced between groups and individuals. To make it easier for the mice to learn that they could escape from the apparatus by entering the hole in training trials with access to the positive reference arm, the outcome of all trials on day 1 (trial 1–5) was exclusively positive. From day 2 onwards a pseudo randomized sequence of trials with access to the positive (+) and negative (−) reference location was used with equal numbers of both locations on each day (day 1: + + + + +, day 2: + − + − − +, day 3: − − + + − +, day 4: + − + −).

The initial interval in the start box was set at 60 s and trials lasted at most 180 s (trial 1+2), 60 s (trial 3–5), or 45 s (trial 6–21). After entering the hole in a training trial with access to the positive reference arm, mice were allowed to stay in their home cage for 30 s until the next trial started or they remained in the home cage in the event that no further trials followed. After a training trial with access to the negative reference arm, the mouse was returned to the start box for 30 s followed by a brief handling (lifting the mouse by its tail) in order to ensure similar handling after a training trial with access to the positive and negative reference arm. Subsequently, the mouse stayed in the start box for another 60 s to begin a new trial or it was returned to its home cage.

To guarantee that mice had learned to discriminate between the positive and negative reference location during training, a learning criterion was defined using the data from day 3, which was the last training day before the cognitive bias test trial. Only data of mice that showed shorter latencies to reach the hole in all training trials with access to the positive reference arm than in all training trials with access to the negative reference arm on day 3 were considered to have reached the criterion and used for later analysis. The criterion was reached by 7 NN mice, 10 CE, and 11 NP mice (Fisher's exact test, p = 0.210).

*Cognitive bias testing:* Mice were tested for their cognitive bias after the last training trial on day 4 in a probe trial. After spending 60 s in the start box, mice gained access to the whole test apparatus with either the near-negative (NN mice), near-positive (NP mice), or central probe arm (CE mice) opened and the four unused arms closed. The probe trial lasted 60 s and the parameters measured were the latency to reach the hole, the latency to reach the arm, and the time spent at the hole.

#### Experiment III

*Apparatus:* The apparatus (Fig. 1B) was similar to that used in experiment I and II with a few modifications. Firstly, a shorter starting corridor was used (15×14×15 cm) and instead of a start box, a dark grey start cylinder (11 cm diameter) was put inside the corridor, in which the mouse was placed before the beginning of each probe or training trial. Secondly, three instead of five equidistantly spaced arms (43×7×15 cm) radiated from the central area: the two reference arms (‘positive’  =  rewarded and ‘negative’  =  aversive), positioned 120° from each other, and one ambiguous probe arm (‘central’) midway between the two reference locations. Instead of sliding doors the unused arms were closed by just reversing them so that their closed end functioned as barrier. Thirdly, in training trials with access to the negative reference arm the air pump was replaced by a compressed air spray. To prevent mice from identifying the outcome of the trial by smelling the compressed air, a small amount of gas was sprayed under the hole at the beginning of each training and probe trial. The movements of the animal were recorded and analyzed by an experienced observer (H.R.) who remained blind to genotype.

*Training:* Mice were trained over a period of 5 days in 25 trials overall to discriminate between the positive ( =  rewarded) and negative ( =  aversive) reference location. Trials on one day were conducted in sessions of 2 or 3 trials each, with the two sessions being interspersed by a break of approximately 3 hours. The position of the positive and negative reference arm was balanced between genotypes and individuals. Again, in order to facilitate overall learning all trials on day 1 (trial 1–5) were exclusively rewarded, while from day 2 onwards a pseudo randomized sequence of trials with access to the positive and negative reference arm was used (day 1: + + + + +, day 2: + − + − + −, day 3: + + − − − +, day 4: + − + − − +, day 5: + −).

After an initial interval of 60 s, the start cylinder was removed and the mouse could freely explore the apparatus. Training trials lasted at most 180 s (trial 1–3) or 60 s (trial 4–25). After entering the hole in a training trial with access to the positive reference arm, mice were allowed to stay in their home cage for 60 s until the next trial started or they remained in the home cage if no further trials followed. After a training trial with access to the negative reference arm, the mouse was returned to the start box for another start interval of 60 s to begin a new trial. If no further trials followed, the mouse was placed in an empty cage for 60 s before being returned to its home cage. This was done to allow a better discrimination between the positive and the negative reference location, as only the positive reference location was intended to be associated with direct access to the home cage.

Once again in experiment III a learning criterion was defined: Only data of mice that showed shorter latencies to reach the hole in all training trials with access to the positive reference arm than in all training trials with access to the negative reference arm on day 3 were used for later analysis (day 4 of training). The criterion was reached by 5 5-HTT +/+ mice, 9 5-HTT +/− mice, and 6 5-HTT −/− mice (Fisher's exact test, p = 0.334).

*Cognitive bias testing:* Mice were tested for their cognitive bias after the last training trial on day 5 in a probe trial. After spending 60 s in the start box, mice could freely explore the test apparatus with the central probe arm opened and the four unused arms closed. The probe trial lasted 60 s and the parameter measured was the latency to reach the hole (latency to reach the arm and percentage of time spent at the hole were not available due to technical difficulties).

### Statistical analysis

All data sets were checked for normal distribution by visual inspection of the histograms as well as by applying the Kolmogorov-Smirnov test with Lilliefors correction. Normally distributed data or data that could be transformed by means of a log transformation were analyzed using one-way analysis of variance (ANOVA) or repeated measures (RM) ANOVA, respectively. For data that were analyzed by means of a one-way ANOVA and that did not meet the assumption of homogeneity of variances, the Welch's adjusted F ratio is reported. For data that were analyzed by means of a RM ANOVA and did not meet the assumption of sphericity, the Greenhouse-Geisser corrected F ratio is reported. Post hoc comparisons for main effects in the one-way ANOVA were conducted using the Tukey-Kramer test for equal variances and the Games-Howell test whenever the homogeneity of variances assumption was violated. Pairwise comparisons in case of interaction effects or significant main effects in the RM ANOVA were performed using independent or dependent samples t-tests, respectively, with sequential Bonferroni correction for multiple comparisons. Data sets that could not be adequately transformed to meet the assumption of normality were analyzed using non-parametric statistics, i.e. Wilcoxon signed-rank test for the comparison of two dependent samples and Kruskal-Wallis test for more than two independent samples.

Statistical significance was set at p≤0.05; p-values p>0.05, ≤0.1 were considered a trend. All tests were calculated using the Software package IBM SPSS Statistics 21 (Release 21.0.0.0, IBM Corporation 2012). Graphs were created using the software SigmaPlot 12.0 for Windows (Build 12.0.0.182, Systat Software, Inc. 2011).

All data underlying the findings described in the present study are fully available in the File S1.

## Results

### Experiment I

#### Training

To assess training performance, data were averaged for each mouse and day and analyzed by means of RM ANOVA with ‘training day’ (day 1 vs. day 2 vs. day 3) as within-subject factor and ‘treatment’ (optimistically- vs. pessimistically-trained mice) as between-subject factor.

**Figure 2:**
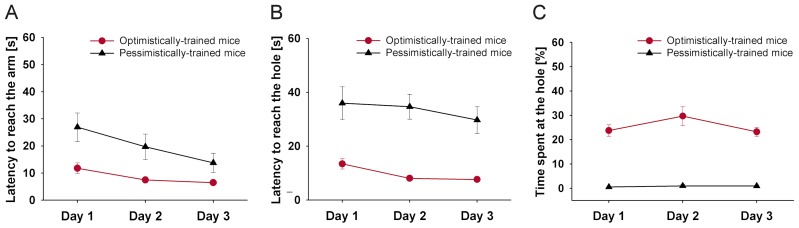
Experiment I: Training. (**A**) Latency to reach the arm, (**B**) latency to reach the hole, and (**C**) percentage of time spent at the hole for mice being confronted with solely positive (optimistically-trained mice, n = 7) or solely negative (pessimistically-trained mice, n = 7) experience across the three days of training. Data are averaged per treatment group and day and are presented as means ±SEM. Day 1: 5 trials, day 2: 6 trials, day 3: 2 trials. See results section for details of statistical analysis.

Optimistically-trained mice showed on average a shorter latency to reach the arm (Fig. 2A, RM ANOVA, F(1, 12)  = 14.299, p = 0.003), a shorter latency to reach the hole (Fig. 2B, RM, ANOVA, F(1, 12)  = 57.066, p<0.001), and spent a higher percentage of time at the hole than pessimistically-trained mice (Fig. 2C, RM ANOVA, F(1, 12)  = 248.520, p<0.001). There was a significant main effect of the training day for the latency to reach the arm (RM ANOVA, F(2, 24)  = 9.011, p = 0.001) as well as for the latency to reach the hole (RM ANOVA, Greenhouse-Geisser corrected F(1.366, 16.387)  = 4.337, p = 0.043). There was no significant training day-by-treatment interaction for either parameter (p>0.1). Thus, there was a decrease in the latency to reach the arm and the hole, regardless of the treatment group (dependent samples t-test, two-tailed, latency to reach the arm: day 1 vs. day 2: t = 2.427, df = 13, p = 0.030, day 1 vs. day 3: t = 4.031, df = 13, p = 0.001, day 2 vs. day 3: t = 2.025, df = 13, p = 0.064; latency to reach the hole: day 1 vs. day 3: t = 2.554, df = 13, p = 0.024). There was neither a significant main effect of the training day nor a significant training day-by-treatment interaction for the percentage of time spent at the hole (p>0.1).

#### Probe trial

**Figure 3:**
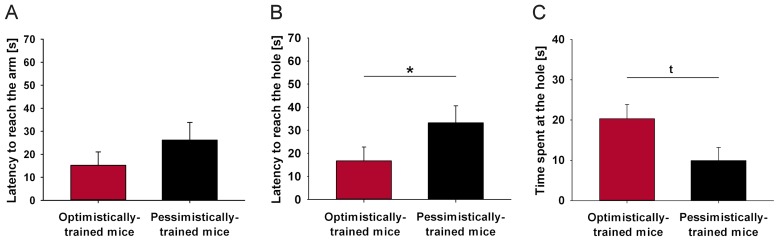
Experiment I: Probe trial. (**A**) Latency to reach the arm, (**B**) latency to reach the hole, and (**C**) time spent at the hole in the central probe arm for mice that have been confronted with solely positive (optimistically-trained mice, n = 7) or solely negative (pessimistically-trained mice, n = 7) experiences during training. Data are presented as means ±SEM. Statistics: ANOVA, main effect of treatment: *p≤0.05, ^t^p≤0.1.

One-way ANOVA with ‘treatment’ as independent variable resulted in no significant difference between optimistically- and pessimistically-trained mice for the latency to reach the arm in the probe trial (Fig. 3A, p>0.1). However, optimistically-trained mice reached the hole significantly faster (Fig. 3B, ANOVA, F(1, 12)  = 4.880, p = 0.047) and tended to spend more time at the hole than pessimistically-trained mice (Fig. 3C, ANOVA, F(1, 12)  = 4.671, p = 0.052).

### Experiment II

#### Training

To assess group performance on each training day, data were averaged for the training trials with access to the positive and negative reference arm for each mouse and day and analyzed by means of RM ANOVA with ‘trial outcome’ (positive vs. negative) as within-subject factor and ‘group’ (near-negative vs. central vs. near-positive) as between-subject factor. As on day 1, the trial outcome was exclusively positive, a one-way ANOVA with ‘group’ as independent variable was used for analysis.

**Figure 4:**
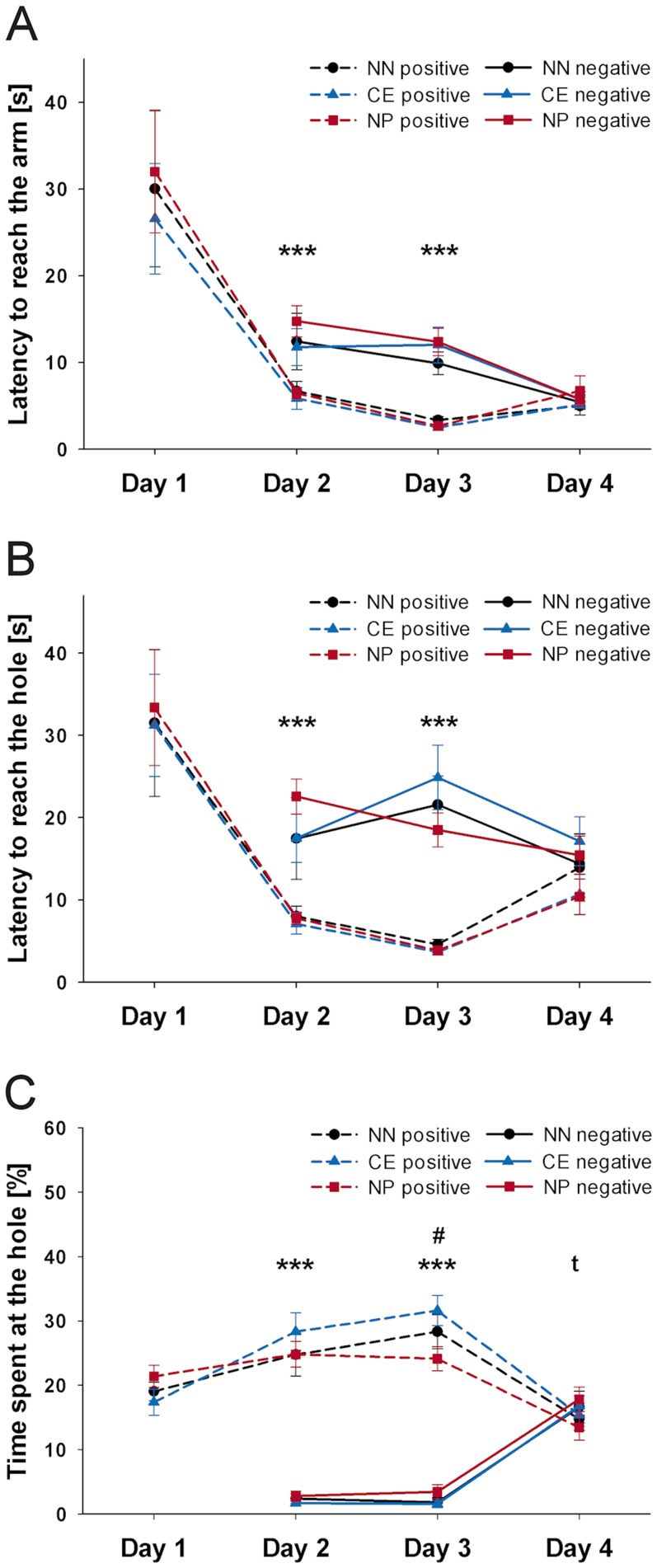
Experiment II: Training. (**A**) Latency to reach the arm, (**B**) latency to reach the hole, and (**C**) percentage of time spent at the hole in positive and negative trials across the four days of training for mice being later confronted with the near-negative (NN, n = 7), central (CE, n = 10), or near-positive (NP, n = 11) probe arm. Data are averaged per trial outcome and day and are presented as means ±SEM. Day 1: 5 positive trials, day 2+3: 3 positive and 3 negative trials, day 4: 2 positive and 2 negative trials. Statistics: day 1: ANOVA; day 2–4: Repeated Measures ANOVA for each day, main effect of trial outcome: ***p≤0.001, ^t^p≤0.1, effect of group-by-trial outcome interaction: ^#^p≤0.1.

There was a significant effect of the trial outcome for the latency to reach the arm on day 2 and day 3 of the training period, with mice reaching the positive reference arm significantly faster than the negative reference arm (Fig. 4A, RM ANOVA, day 2: F(1, 25)  = 31,630, p<0.001; day 3: F(1, 25)  = 169.597, p<0.001). Moreover, mice reached the hole in the positive reference arm significantly faster than the hole in the negative reference arm (Fig. 4B, RM ANOVA, day 2: F(1, 25)  = 38.354, p<0.001; day 3: F(1, 25)  = 86.695, p<0.001) and spent a higher percentage of time there (Fig. 4C, RM ANOVA, day 2: F(1, 25)  = 224.446, p<0.001; day 3: F(1, 25)  = 349.838, p<0.001). Surprisingly, there was no significant effect of the trial outcome for the latency to reach the arm or the latency to reach the hole on day 4 of training (p>0.1) and mice even tended to spend a higher percentage of time at hole in the negative reference arm compared to the hole in the positive one on this day (RM ANOVA, F(1, 25)  = 3.567, p = 0.071). However, comparing only the last rewarded and last aversive training trial immediately before the cognitive bias test on day 4 by means of a Wilcoxon signed-rank test, mice discriminated correctly between the two reference locations: They reached the arm and the hole significantly faster in the positive than in the negative reference location and also investigated the hole in the positive reference arm significantly longer compared to the hole in the negative one (Wilcoxon test, two-tailed, latency to reach the arm NN mice: Z = −2.366, p = 0.018, CE mice: Z = −2.803, p = 0.005, NP mice: Z = −2.934, p = 0.003; latency to reach the hole NN mice: Z = −2.371, p = 0.018, CE mice: Z = −2.397, p = 0.017, NP mice: Z = −2.934, p = 0.003; percentage of time spent at the hole NN mice: Z = −2.366, p = 0.018, CE mice: Z = −2.803, p = 0.005, NP mice: Z = −2.934, p = 0.003).

Regarding group differences, there were no significant effects of the group on any day of the training period for any parameter (p>0.1). However, there was a trend for a group-by-trial outcome interaction for the percentage of time spent at the hole on day 3 (RM ANOVA, F(2, 25)  = 3.352, p = 0.051): Although mice of all three groups spent more time at the hole in the positive than the negative location (dependent samples t-test, two-tailed, NN: t = 10.523, df = 6, p<0.001, CE: t = 14.856, df = 9, p<0.001, NP: t = 8.698, df = 10, p<0.001), CE mice spent a higher percentage of time at the hole location than NP mice (independent samples t-test, two-tailed, t = 2.519, df = 19, p = 0.021).

#### Cognitive bias testing

**Figure 5:**
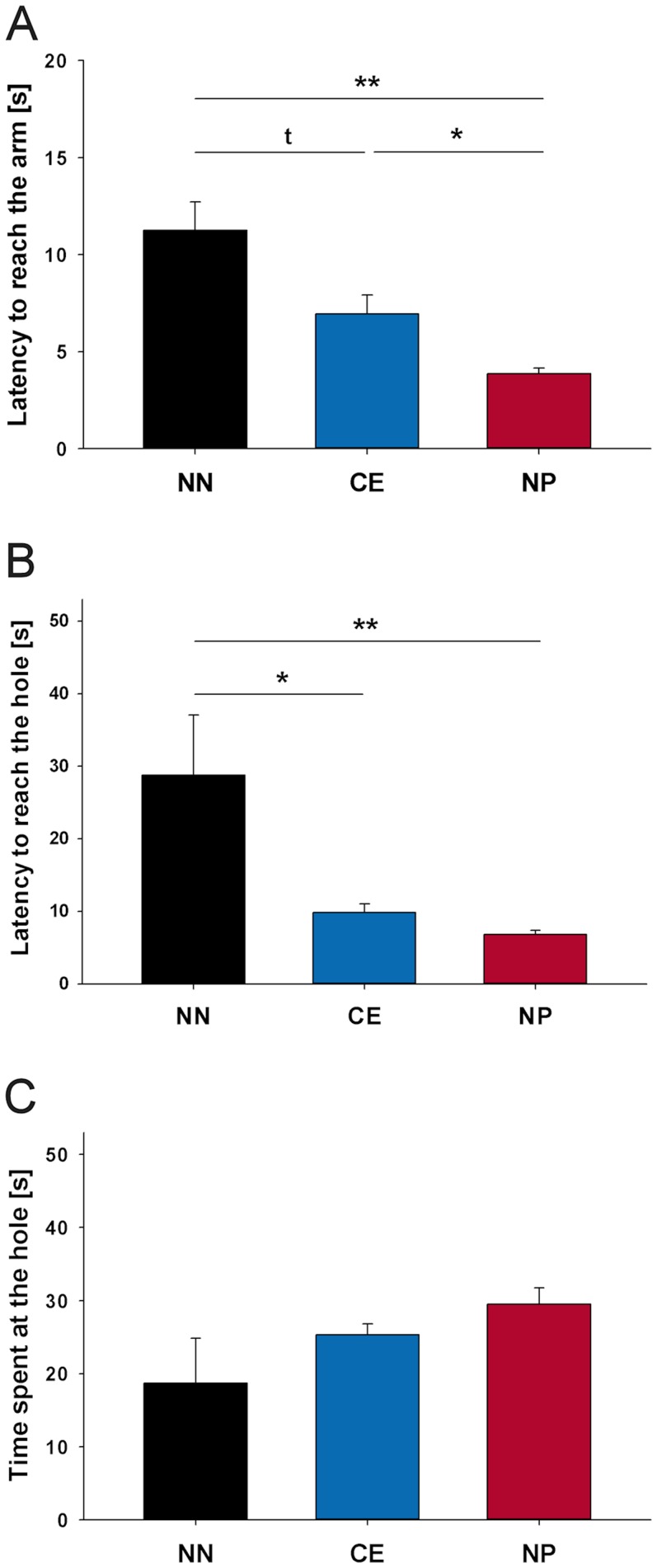
Experiment II: Cognitive bias test. (**A**) Latency to reach the arm, (**B**) latency to reach the hole, and (**C**) time spent at the hole for mice being confronted with either the near-negative (NN, n = 7), central (CE, n = 10), or near-positive (NP, n = 11) probe arm. Data are presented as means ±SEM. Statistics: ANOVA, post hoc testing: Games-Howell test, **p≤0.01, *p≤0.05, ^t^p≤0.1.

One-way ANOVA with ‘location’ as independent variable revealed a significant effect of the location of the ambiguous arm for the latency to reach the arm (Fig.5A, ANOVA, Welch's F(2, 10.721)  = 15.071, p = 0.001) as well as for the latency to reach the hole (Fig. 5B, ANOVA, Welch's F(2, 12.626)  = 9.243, p = 0.003). Mice reached the near-positive arm significantly faster than the near-negative arm (Games-Howell test, p = 0.005) and the central arm (Games-Howell test, p = 0.030) and tended to reach the near-negative arm later than the central arm (Games-Howell test, p = 0.075). Moreover, mice hesitated significantly longer to reach the hole in the near-negative location compared to the central location (Games-Howell test, p = 0.040) and the near-positive location (Games-Howell test, p = 0.009). There was no effect of the location for the time spent at the hole (Fig. 5C, p>0.1).

### Experiment III

#### Training

For the training analysis on days 2–4, data were averaged for the training trials with access to the positive and negative reference arm for each mouse and analyzed by means of RM ANOVA with ‘trial outcome’ (positive vs. negative) as within-subject factor and ‘genotype’ (5-HTT +/+ vs. 5-HTT +/− vs. 5-HTT −/−) as between-subject factor on each day. Again on day 1 the trial outcome was exclusively positive and, therefore, a one-way ANOVA with ‘genotype’ as independent variable was used. As training data sampled on day 5 were not normally distributed, differences were evaluated by means of the Wilcoxon signed-rank test for the analysis of the influence of the trial outcome and by means the Kruskal-Wallis test for the evaluation of genotype differences.

**Figure 6:**
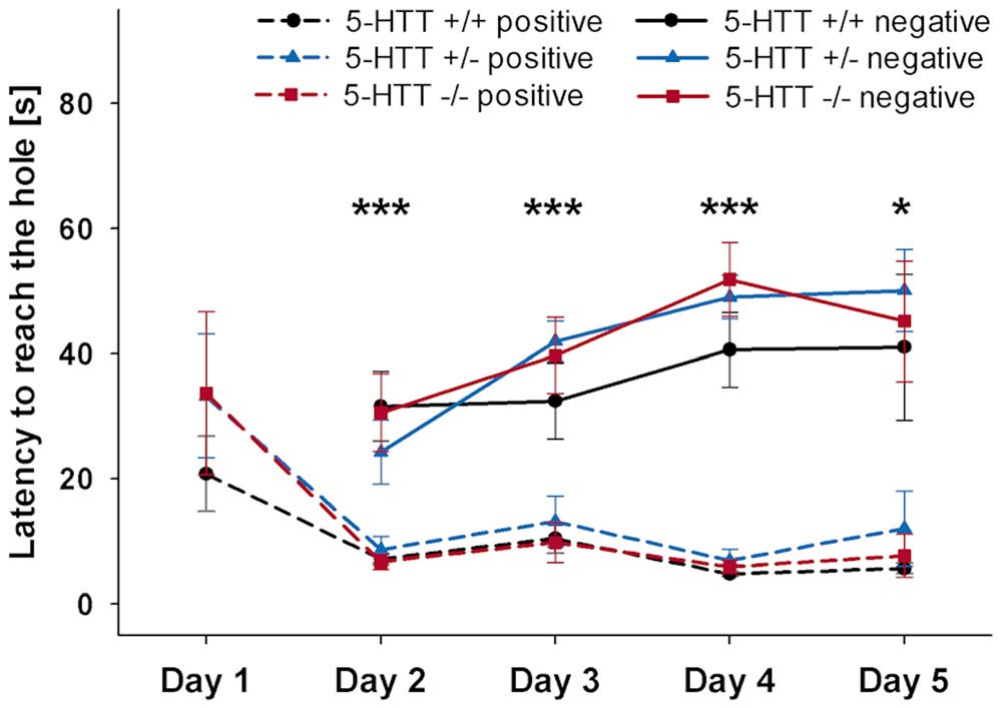
Experiment III: Training. Latency to reach the hole in positive and negative trials across the five days of training days for 5-HTT +/+ (n = 5), 5-HTT +/− (n = 9), and 5-HTT −/− mice (n = 6). Data are averaged per trial outcome and day and are presented as means ±SEM. Day 1: 5 positive trials, day 2+3+4: 3 positive and 3 negative trials, day 5: 1 positive and 1 negative trial. Statistics: day 1: ANOVA; day 2–4: Repeated measures ANOVA for each day, main effect of trial outcome ***p≤0.001; day 5: Wilcoxon signed-rank tests (two-tailed), *p≤0.05 within each genotype.

There was a significant effect of the trial outcome for the latency to reach the hole on each day of the training period with mice reaching the hole significantly faster in the positive than in the negative reference location (Fig. 6, RM ANOVA, day 2: F(1, 17)  = 71.079, p<0.001; day 3: F(1, 17)  = 48.764, p<0.001; day 4: F(1, 17)  = 206.661, p<0.001; Wilcoxon test, day 5: 5-HTT +/+: Z = −2.023, p = 0.043, 5-HTT +/−: Z = −2.555, p = 0.011, 5-HTT −/−: Z = −2.201, p = 0.028). There were no genotype or genotype-by-trial outcome interaction effects for the latency to reach the hole on any day of the training period (p>0.1).

#### Cognitive bias testing

**Figure 7:**
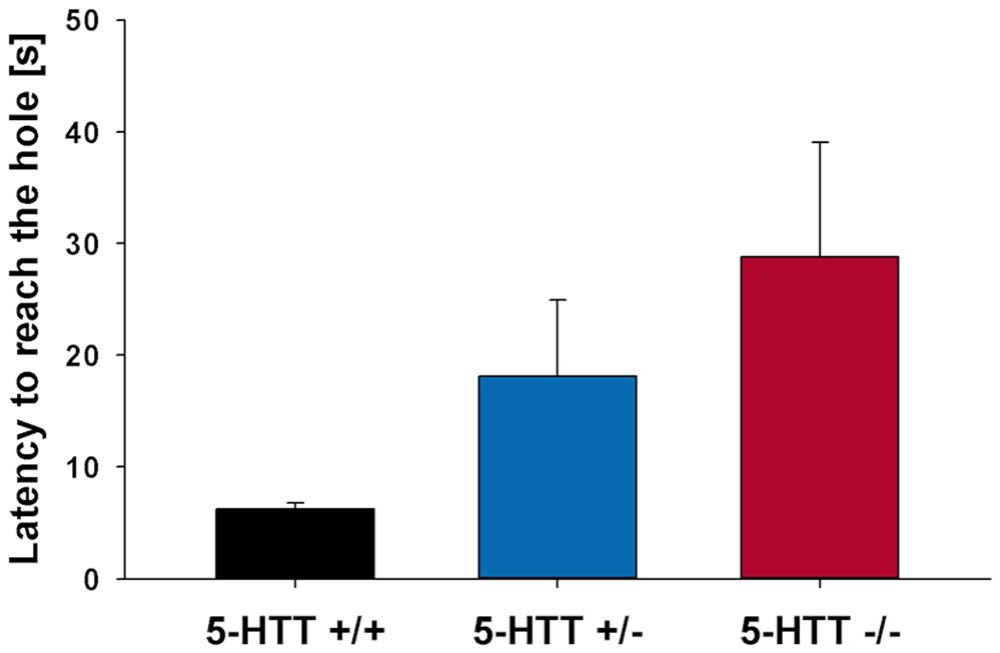
Experiment III: Cognitive bias test. Experiment III: Cognitive bias test. Latency to reach the hole in the central probe arm for 5−HTT +/+ (n  =  5), 5−HTT +/− (n  =  9), and 5−HTT −/− mice (n  =  6). Data are presented as means ± SEM.

One-way ANOVA with ‘genotype’ as independent variable revealed a non-significant trend for a genotype effect for the latency to reach the hole (Fig. 7, ANOVA, F(2, 17)  = 2.668, p = 0.098) with highest values in 5-HTT -/- mice and lowest values in 5-HTT +/+ mice.

## Discussion

Affect-induced cognitive bias has proven to be a promising new measure of animal emotion [1]. Three experiments were conducted to establish a reliable method to assess cognitive bias in mice, using a spatial judgment task. Our aims were, first, to prove that the test paradigm can assess different expectations about the outcome of an unfamiliar stimulus, second, to evaluate spatial location as a discriminatory stimulus, and, third, to apply the task in a pilot study with 5-HTT knockout mice - a well-established mouse model for the study of anxiety- and depression-related behavior.

The present study used an exploratory research design to lay the groundwork for more-complete cognitive bias research in mice, thus several procedural changes were introduced over the course of the three experiments to optimize the test protocol. As even minor changes in the experimental setting can have strain-dependent consequences for behavioral outcomes [52], [53], this should be noted when comparing the results of three experiments with one another.

### Experiment I

During the whole training period, optimistically- and pessimistically-trained mice differed significantly from each other in their behavior towards the two reference locations, with optimistically-trained mice reaching the positive locations faster compared to pessimistically-trained mice that reached the negative locations slower or not at all. This confirms the use of access to the home cage as reinforcement and the confrontation with an air-puff as punishment, and demonstrates their capacity to induce a clearly discriminable behavioral response in mice. The possibility to escape from the brightly lit test apparatus into the home cage via a hole takes advantage of the propensity of mice to find and escape through small holes and addresses the natural preference of rodents for dark and protected environments over brightly lit and less protected areas [54]–[56]. Therefore, the reinforcer used here may represent a valuable alternative to the frequently used food reinforcers, which are prone to the effects of food motivation or food reward valuation [1] and often require a preceding period of food deprivation. At the same time, the air-puff stimulus which is known to induce fear-related reactions such as a startle response and avoidance behavior in rodents [57]–[59], may be a suitable alternative punishment to frequently used foot-shocks in paradigms where aversive memory formation is required [59]. Nevertheless, there might have been some habituation to the air-puff over the course of the training period: Though significantly different between the two treatments in absolute values, the latency to reach the arm and the latency to reach the hole decreased over the course of training, regardless of the actual treatment group. To reduce habituation effects, future studies may apply a stronger and more defined air-puff, than was possible here with the manually-operated air-pump, e.g. by using a small electrical air compressor or tanks of compressed air.

Confronted with an unfamiliar probe location, optimistically- and pessimistically-trained mice showed the predicted behavioral differences: Optimistically-trained mice reached the unfamiliar probe location faster and tended to spend more time exploring it compared to pessimistically-trained mice. Thus, mice which learned to expect something positive within the maze show a more positive judgment of an unfamiliar stimulus of intermediate spatial location than mice which learned to expect something negative. This confirms that optimistic- and pessimistic-like biases in mice can be assessed by their behavior towards an unfamiliar stimulus. It should be mentioned, however, that the findings in the probe trial could also have resulted from treatment-induced differences in the exploratory drive of the animals. In future studies, independent measures of exploration should be assessed that would help to rule out such effects unequivocally [1].

### Experiment II

Mice that passed the individual learning criterion discriminated correctly between the positive and the negative reference location already on the second day of training, which was the first day on which both training cues were presented in parallel: There was a significant preference for the positive location, indicated by shorter approach latencies and a higher percentage of time spent exploring it. Strikingly, after showing a significant preference for the positive location during the first three days of training, this preference disappeared on the fourth day of training, when mice approached and explored both locations to a similar degree. Two explanations for this finding might be possible: Firstly, mice might have habituated to the air-puff upon repeated presentation [60], [61], while at the same losing motivation to escape from the apparatus, as they perceived that there were no major threats which require a quick escape [62]. The second explanation refers to an incomplete memory formation on the fourth day of training. On that day the average training performance of the mice was calculated on the basis of 2 positive and 2 negative trials each, while on day 2 and 3 it was based on 3 positive and 3 negative trials, respectively. In the case that mice were not able to correctly recall the position of the reference locations from the previous day, a random choice on the first trial would more strongly affect the overall score than would be the case on the other days, for which a correct discrimination could be proven. Consequently, more training days or more trials on each training day might be necessary to induce a stable place discrimination that persists from one day to another. The implementation of more trials on one day may further help to optimize the individual learning criterion which based on a descriptive analysis of the performance of the mice in positive and negative trials on the day before cognitive bias testing was performed. Future studies may include a number of trials which would allow the use of an individual learning criterion that can be checked statistically, e.g. five positive and five negative trials per day. This criterion could then be required to be reached by the mice on at least two consecutive days, before moving on to the probe trial. Furthermore, it might be advisable to include a visual landmark within the apparatus to facilitate orientation within the maze [18], [21]. These measures may not only enhance the learning progress of the mice, but may further reduce the number of individuals that have to be excluded from the experiment because they fail to meet the individual learning criterion.

Notwithstanding the absence of a clear preference for the positive location on day 4 as a whole, mice showed a clearly discriminable behavioral response without any group differences in the last positive and the last negative training trial immediately before the cognitive bias test trial on that day. This indicates that despite the described limitations the learning progress of the mice was sufficient to reliably test for their ambiguous cue interpretation.

In line with our hypothesis, mice showed a graded response to the three probe locations, reflecting their level of ambiguity: The near-negative location was approached most slowly, the near positive location was approached most rapidly, and the central location was reached at intermediate values. Similar to findings in rats [19], the judgment of the mice about an ambiguous probe location, therefore, depends on its spatial location within the maze, with the central probe arm provoking the highest level of ambiguity. This confirms that spatial location functions as a discriminatory stimulus in laboratory rodents [36] and can be used to assess judgment biases in mice. Please note: In view of the inconsistencies during the training period mentioned above, it might be advisable to repeat the experimental approach with an optimized test design to definitively prove the reproducibility and external validity of the finding.

Although the central probe location appears to be most suitable for an intuition-guided interpretation, also the near-negative and the near-positive probe location may help to reveal a more detailed picture of the emotional state of an animal. More specifically, differences in the interpretation of a near-negative or a near-positive cue may provide information on whether the animal shows a decreased anticipation of a positive event (difference in the judgment of the near-positive cue) or an increased anticipation of a negative event (difference in the judgment of the near-negative cue) [1], [37]. While the former may indicate a depression-like state, the latter is generally associated with anxiety, thus individuals experiencing the same-valence affective states can show different types of cognitive bias [63]–[65].

### Experiment III

Mice of all three 5-HTT genotypes that had passed the individual learning criterion discriminated correctly between the positive and the negative reference location throughout the whole training period. Moreover, although a relatively high number of individuals had to be excluded from the experiments as they did not meet the individual learning criterion, the number of excluded animals also did not differ between the genotypes (see experiment II for discussion on improvement of training success). The absence of any genotype-dependent differences regarding the learning process confirms the earlier findings of a comparable spatial learning capacity of 5-HTT +/+, 5-HTT +/−, and 5-HTT −/− mice in a Barnes maze test with access to the home cage as reinforcer [50]. Additionally, the results suggest that neither the reduced general activity nor the decreased exploratory activity in 5-HTT −/− mice [44], [47]–[49], [66] influenced the outcome of the following cognitive bias testing. If there were any effects of global activity or exploration deficits on the performance of the mice, those would likely have influenced not only the response to the ambiguous probe cue but also that to the training cues [1].

When comparing the latency to approach the ambiguous probe location, there was a non-significant trend for a genotype effect with 5-HTT −/− mice hesitating the longest and 5-HTT +/+ mice hesitating the shortest to approach the ambiguous hole. This potential positive outcome is an interesting hint for a negative response bias in mice with abolished 5-HTT function, which are characterized by an increased anxiety- and depression-like phenotype, and, therefore, are in a putatively negative affective state [44]–[48]. However, as the result did not reach statistical significance, larger scale studies with an optimized test design are needed to confirm its external validity. If a replicate experiment yields a significant pessimistic response bias in 5-HTT −/− mice, this would substantiate that the test paradigm is suitable to assess judgment biases resulting from the emotional state of an individual. In general, a pessimistic response bias in 5-HTT −/− mice would fit well with findings in humans, where reduced 5-HTT expression is generally associated with a negative attentional bias, namely an increased reactivity and attention towards negatively-valenced information [67]–[71].

In this context, we would like to encourage the use of 5-HTT +/− mice in the study of 5-HTT-related changes in cognitive bias. 5-HTT +/− mice displayed an intermediate phenotype compared to the performance of 5-HTT +/+ and 5-HTT −/− mice in the probe trial. This suggests a more pessimistic judgment than in wildtype mice, although not as negative as mice with the complete loss-of-function mutation. Since 5-HTT +/− mice are generally less affected by genetically-induced exploration deficits and hypoactivity than 5-HTT −/− mice, their incorporation may enable a clearer interpretation of the animals’ behavior in the task [72]. Moreover, 5-HTT +/− mice can develop signs of impaired emotion regulation in combination with stressful live events [51], [73], resembling 5-HTT genotype-by-environment interactions observed in human studies [74], [75]. Consequently, 5-HTT +/− mice might be of particular relevance when studying how cognitive bias can be modulated by certain environmental factors in combination with a genetic predisposition for anxiety and depression.

In contrast to several other cognitive bias tests, the present study uses a one-trial testing procedure in order to avoid confounding learning effects associated with repeated presentation of the unrewarded ambiguous location. Hence, it cannot be excluded that the single test trial may be biased by contextual features (e.g. sudden noise, individual arousal). Although appropriate sample sizes as well as strict control of the laboratory environment may reduce such confounding effects to a minimum, studies on cognitive bias may generally benefit from combining findings on ambiguous cue interpretation with tests on other behavioral or physiological indicators of animal emotion (e.g. tests for anxiety- or depressive-like behavior, assessment of HPA axis activity). Demonstrating that a genetic or environmental manipulation produces behavioral alterations in various tests for emotional arousal *and* emotional valence, would provide strong support for a true effect and similarly enable a detailed characterization of the emotional profile of an individual [76].

### Conclusions

This study set out to establish a novel spatial judgment task to assess affect-induced cognitive bias in mice. After demonstrating that mice with varying anticipation of future positive or future negative experiences differ in their behavior towards an unfamiliar cue, and that spatial location functions as a discriminatory stimulus, there was a hint for 5-HTT genotype inducing a response bias in mice that would fit the depressive-like phenotype of the 5-HTT knockout mouse model. Therefore, the task described may serve as a valuable base for evaluating cognitive bias in mice, which in turn can give indication on the emotional state of an animal. Since cognitive affective biases are a key feature of depression and anxiety disorders in humans, the opportunity to study them in the mouse may help to reveal underlying biological processes and provide new approaches for therapeutic treatment. In addition to its translational value, the test may also hold great potential for animal welfare research, where better knowledge and accurate assessment of animal emotion is equally indispensable.

## Supporting Information

File S1 **Raw data.** Excel file containing all data underlying the findings described in the article with sheet one containing the data of experiment I, sheet two containing the data of experiment III, and sheet three containing the data of experiment III.
(XLSX)
